# The Boston Dental College

**Published:** 1899-09

**Authors:** 


### THE BOSTON DENTAL COLLEGE.

This college, which has been the recipient of much uncalled-for criticism, founded mainly upon personal feeling, has united its interests with Tufts College, and it became, on July 1, the Dental Department of that college. The medical lectures will be given in the Tufts Medical School. It is the intention of the college authorities to erect, in the near future, a building for the use of the dental school.

The laws of Massachusetts make it impossible for any but medical schools to receive subjects for dissection. This very unjust discrimination against dental colleges has involved the Boston college in a difficulty, for the National Association of Dental Faculties has a rule requiring all colleges connected with it to have facilities for the study of anatomy by dissection.

This rule is gradually forcing all dental colleges to become part of either medical colleges or universities, and the day is not far distant when the independent dental school will have passed into history. This is simply a step in the direction of a final merging of the dental profession into that of the medical and the eventual obliteration of the D.D.S. as a distinct degree. This may not be an agreeable thought to some, but the indications all point in that direction. The present changes taking place are for the benefit of dentistry, but whether the final absorption will result in a higher degree of practical excellence is a question as yet unsolved.

